# Prevalence of hepatitis E virus and porcine caliciviruses in pig farms of Guizhou province, China

**Published:** 2011-06-01

**Authors:** Quan Shen, Rongqing Ren, Wen Zhang, Zhibiao Yang, Shixing Yang, Yan Chen, Li Cui, Xiuguo Hua

**Affiliations:** 1 School of Agriculture and Biology, Shanghai JiaoTong University, Shanghai, China; 2 Food Animal Health Research Program, Ohio Agricultural Research and Development Center, The Ohio State University, Wooster, Ohio, USA; 3 School of Medical Technology, Jiangsu University, Zhenjiang, Jiangsu, China; 4 Guizhou Veterinary Research Institute, Guizhou Academy of Agricultural Sciences, Guiyang, Guizhou, China

**Keywords:** Hepatitis E virus, Porcine, Caliciviruses, Genetic diversity

## Abstract

**Backgroud:**

Hepatitis E virus (HEV) and caliciviruses are enteric pathogens of humans and animals, and pigs have been considered an important reservoir of these viruses.

**Objectives:**

The aim of this study is to determine the infection rates of HEV and caliciviruses (sapovirus [SaV] and norovirus [NoV]) in different age groups of pigs in Guizhou province, China, and characterize the strains that are prevalent in this mountainous area.

**Materials and Methods:**

A total of 209 stool samples from healthy pigs of different ages were collected from 6 pig farms in Guizhou province from May to June 2009 and tested for HEV, SaV, and NoV by reverse-transcription polymerase chain reaction (RT-PCR).

**Results:**

The overall prevalence of porcine HEV and porcine SaV was 6.7% (15/209) and 1.0% (2/209), respectively. No NoV was detected. The prevalence rates of porcine HEV infection were 15.4% in piglets aged < 1 mon (4/26), 6.8% in piglets aged between 1 and 4 mon (3/44), 12.5% in piglets aged ≈ 4 mon (6/48), and 1.1% in sows aged between 6 and 14 mon (2/91). Porcine SaV was detected only in piglets (7.7%, 2/26). All 10 HEV isolates belonged to genotype 4, clustering with a human HEV strain (AF103940) from an adjacent province.

**Conclusions:**

This is the first report on the existence of porcine SaV in swine in Guizhou province, China. The clustering of the porcine HEV isolates with a human strain suggests cross-species transmission between swine and humans in this area.

## 1. Background

Hepatitis E virus (HEV) is a member of the genus Hepevirus and has a 7.2 kb positive-sense RNA genome that contains 3 open reading frames (ORFs) [1]. Based on a sequence analysis, HEV strains have 4 major genotypes (genotype1-4). HEV is considered to be a zoonotic agent, and researchers have suggested that swine is a principal reservoir of HEV that infects humans [2][3][4][5][6]. The family Caliciviridae is divided into 4 genera: Norovirus (NoV), Sapovirus (SaV), Lagovirus, and Vesivirus. NoV and SaV are common causes of gastroenteritis in humans and have been detected in several animal species- SaV in swine and mink and NoV in swine, cattle, mouse, lion, and dog [7]. Porcine calicivirus infections have been reported in industrialized and developing countries [8][9].

Porcine SaV and porcine NoV are considered zoonotic agents due to the genetic and antigenic similarities between porcine and human strains and the occurrence of recombination [10][11][12][13][14][15]. Porcine HEV and porcine caliciviruses are transmitted primarily through the fecal-oral route and are excreted in feces. Some reports have shown that genotype 4 HEV is transmitted freely between swine and humans in eastern and southern China; however, other studies have generated contrasting results for other areas of China [6][16][17]. We have shown that porcine caliciviruses exist on pig farms in eastern China and that porcine SaV causes diarrhea outbreaks in piglets. The infection rates for porcine SaV and NoV in this area are 0.9% and 0.2%, respectively. The prevalence of HEV in stool samples of Chinese swine is 9.6% to 26.1%, and the overall seroprevalence in humans is 43% in rural communities of southern China [16][17][18]. However, no coinfection of HEV and caliciviruses has been observed in humans or animals.

## 2. Objectives

Little is known about the genetic diversity of the porcine HEV and caliciviruses that circulate in southwestern China, particularly in Guizhou province, a developing mountainous area. Therefore, the aim of this study was to determine the infection rates of HEV and caliciviruses in various age groups of pigs and characterize the strains that are prevalent in this area.

## 2. Materials And Methods:

A total of 209 stool samples from healthy pigs of various ages were collected randomly from 6 middle- or large-scale pig farms (200-2000 sows each) in Guizhou province from May to June 2009. Fresh stool samples were collected and prepared immediately as 10% (w/v) suspensions in PBS (0.01 M phosphate, pH 7.2-7.4, 0.15 M NaCl, 0.1% DEPC). RNA was extracted from 200 ul of 10% fecal suspension with TRIzol (Invitrogen, USA) per the manufacturer's instructions after low-speed centrifugation. RNA pellets were dissolved in 25 ul of RNase-free water, and reverse-transcription was performed immediately. Two sets of primers were used to detect human and porcine caliciviruses, as reported [9]. Universal HEV primers were used to detect all 4 HEV genotypes as described [19]. Reverse-transcription was performed in a 10-ul reaction containing 2 ul 5×RT buffer, 0.5 ul (200 units) AMV reverse transcriptase (TaKaRa, Japan), 1 ul (25 mM) primer, and 0.5 µg extracted RNA at 42°C for 1 hr. The parameters for the PCR have been reported (2, 20).

**Figure 1:**
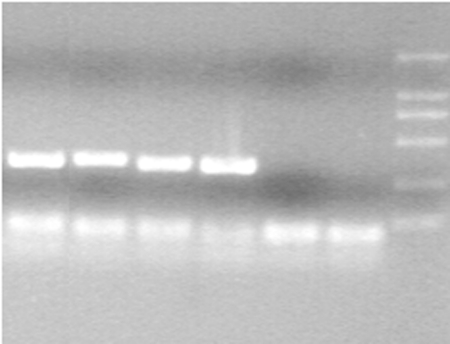
PCR products for HEV and SaV. Lane 1 to lane 6: negative control for HEV, negative control for SaV, positive control for HEV, HEV-positive sample, positive control for SaV, SaV-positive sample

The PCR products were analyzed on a 1.5% agarose gel, stained with ethidium bromide (0.5 ug/ml), and visualized under UV light (Figure 1). The expected DNA bands were purified with the AxyPrep DNA Gel Extraction Kit (Axygen, USA) and cloned into the pMD18-T vector (TaKaRa, Japan). The inserts were sequenced on a DNA analyzer (Applied Biosystems 3730; Invitrogen, USA). To avoid contamination, negative and positive controls were added from RT-PCR step to nucleotide sequencing. After multiple alignment with CLUSTAL W (version 1.4), MEGA, version 4.0 was used to construct phylogenetic trees of the HEVs and SaVs by bootstrap analysis (1000 repeats).

## 3. Results

Porcine HEV and porcine SaV were detected in 83% (5/6) and 33% (2/6) of the farms in this study, respectively. No NoV was detected. Moreover, there was no pig that was coinfected with HEV and SaV. All specific PCR bands were TA-cloned and sequenced. Viral strains with identical nucleotide sequences were considered a unique strain in the phylogenetic analysis, yielding 10 strains with distinct sequences of HEV and 2 unique strains of SaV. The overall prevalence of porcine HEV and porcine SaV was 6.7% (15/209) and 1.0% (2/209), respectively. The prevalence rates of porcine HEV infection were 15.4% in piglets aged < 1 mon (4/26), 6.8% in piglets aged between 1 and 4 mon (3/44), 12.5% in piglets aged ≈ 4 mon (6/48), and 1.1% in sows aged between 6 and 14 mon (2/91). The prevalence of HEV in piglets and finisher pigs aged ≈ 4 mon) was significantly higher than in other age groups (P < 0.01). Porcine SaV was detected only in piglets (7.7%, 2/26) (Table 1).

**Table 1:** Frequency of HEV and SaV by RT-PCR in stool samples in pigs of different age

**Age** (mo)	**Samples tested **(No.)	**Positive samples for HEV** [No. (%)]	**Positive samples for SaV **[No. (%)]
**Sow **(6-14)	91	1 (1.1%)	0 (0.0%)
**Finisher **(4)	48	6 (12.5%)	0 (0.0%)
**Nursery pig **(1-2)	44	3 (6.8%)	0 (0.0%)
**Piglet **(< 1)	26	4 (15.4%)	2 (7.7%)
**Total **	209	14 (6.7%)	1.0%

**Figure 2:**
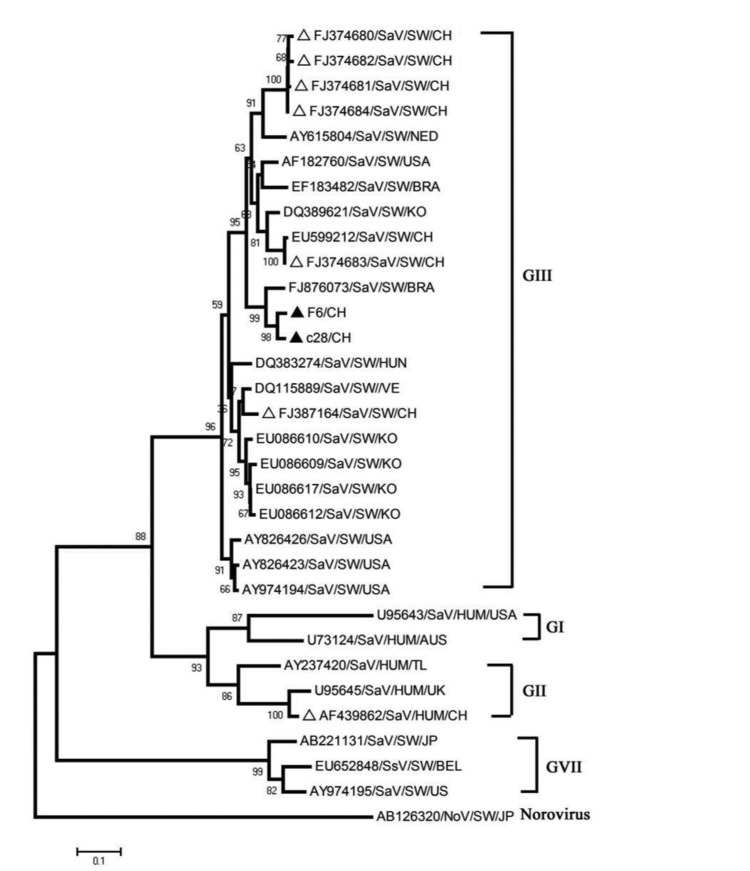
Neighbor-joining tree of the phylogenetic relationship between HEV strainss based on a 304-nt fragment of ORF2. Bootstrap values, expressed as percentages of 1000 replications, are given at the branch point. The 10 newly identified HEV strains

**Figure 3:**
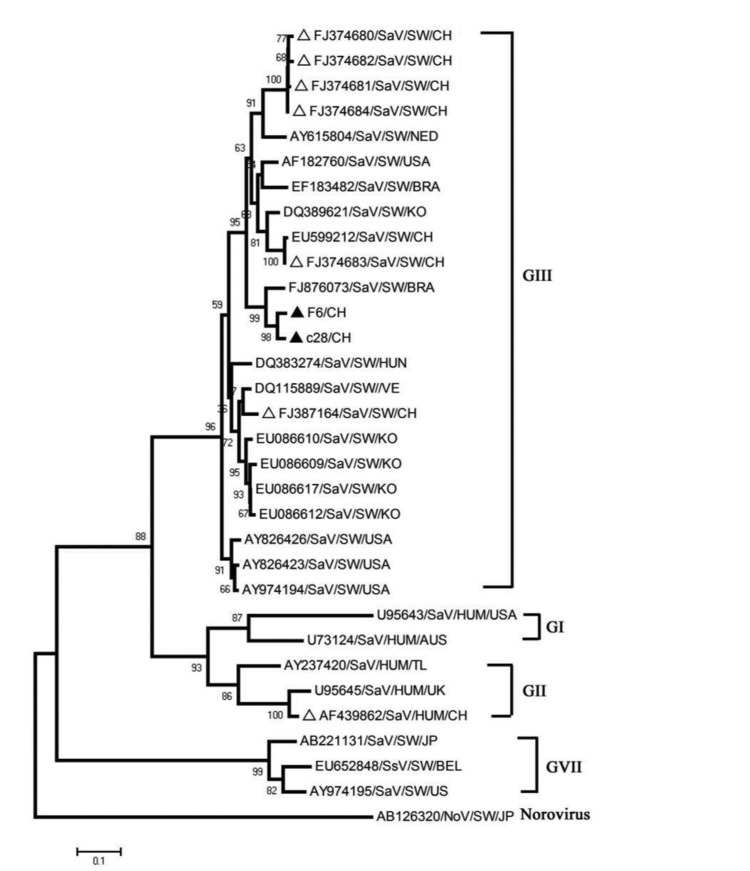
Neighbor-joining tree of the phylogenetic relationship between SaV strains based on a 309-nt fragment of the RNA-dependent RNA polymerase gene. Bootstrap values, expressed as percentages of 1000 replications, are given at the branch points. Genbank accession numbers for the reference strains are marked at each branch points. The 2 newly identified SaV strains (Genbank accession numbers: HQ292717 for C28/CH; HQ292716 for F6/CH) are marked by filled triangles, and other Chinese SaV strains are marked by unfilled triangles.

By phylogenetic analysis, all 10 HEV strains belonged to genotype 4 (Figure 2). They shared 90% to 99% nucleotide homology and 92% to 94% homology with a human HEV strain (AF103940), which was isolated from the Guangxi Zhuang Autonomous Region, which neighbors Guizhou province [21]. The 2 porcine SaVs belonged to SaV GIII and shared 96% nucleotide homology (Figure 3). They clustered with a Brazilian strain (FJ876073), sharing 91% nucleotide homology with it. Yet, they shared a maximum of 84% nucleotide identity with the strain (FJ374683) from eastern China. Notably, they shared 84% nucleotide homology with a human SaV from China (AF439862).

## 5. Discussion

HEV causes outbreaks in developing countries and sporadic hepatitis in industrialized countries [22][23]. Swine is considered a principal reservoir of the HEV that infects humans in regions of China [6]. HEV RNA has been detected in swine stool samples in over 16 provinces and regions of China. In this study, 14 of 209 (6.7%) fecal samples were positive for HEV RNA Some studies have observed that growing pigs have the highest HEV shedding rates, whereas we noted that piglets had the highest rate. The climate, presence of a river, and water supply and management can affect the prevalence of HEV [24]. Therefore, we posited that our results were attributed to the specific climate, location, and sanitary conditions of the farms in our study. The lower overall prevalence of HEV might be due to the sows' previous exposure to HEV and the resulting acquired immunity and lower infection rate.

Additional research should be performed to determine any differences in the characteristics of infection in this mountainous area. Evidence is accumulating that HEV is zoonotic and that pigs are considered a major reservoir of human infection. By phylogenetic analysis of its partial or entire genome, genotype 4 HEV is transmitted freely between humans and swine in eastern China and some provinces of southern China [6][25]. Nevertheless, our analysis of HEV infection in central China revealed no evidence of cross-species transmission between humans and swine [24]. In our study, all 10 HEV isolates belonged to genotype 4, clustering with a human strain (AF103940) from an adjacent province contains both Guizhou and Guangxi Zhuang Autonomous Region, which suggests that cross-species transmission between swine and human occurred in this area.

Although genotype 3 HEV was detected in provinces of China, including Shanghai, Henan, Anhui, and Zhejiang, genotype 4 is the principal strain in China [24]. All 10 stains belonged to genotype 4, and no genotype 3 strains were detected. Genotype 3 strains are more sensitive than genotype 4 to strict sanitation procedures, such as sterilization of antibiotic-laden environments and the adoption of an all-in/all-out management system. The genotype 3 strains might have been new ‘immigrants' to China that are unable to survive such unfavorable conditions, resulting in decreased infection compared with better adapted, ‘native' HEV genotype 4 strains [25]. These hypotheses explain why genotype 4 strains are more prevalent and are emerging as the chief strains in China.

Porcine calicivirus infections have been reported in industrialized and developing countries. SaV and NoV are common in animals, and some of them are genetically closely related to human strains [12][26]. Moreover, more potential recombinant strains of SaV and NoV have been reported recently [12][27][28]. The detection of SaV and NoV in many countries and various species implicates a potential zoonotic risk of cross-species infections. We reported that porcine SaV and NoV infection exists in pigs in eastern China [9][29]. Two porcine SaV strains were observed in the current study, both of which were isolated from piglets. The overall prevalence rate for SaV was 1.0%, which is similar to that in eastern China (0.9%) but significantly lower than in Brazil (30.1%), Venezuela (17.6%), and the USA (62%) [9][27][28][29][30][31][32]. NoV infection is seasonal, peaking in winter (from October to April), most notably in February and March[33]. Not finding NoV infection in our study might be partially due to this epidemic characteristic of NoV.

Or phylogenetic analysis indicated that the 2 porcine SaV isolates clustered with a Brazilian isolate, suggesting that they have a common genetic origin with the Brazilian strain rather than with Chinese isolates. Moreover, porcine SaVs exist in not only eastern China but also southwestern China, although the infection rate there is low. The relationship between human calicivirus and porcine calicivirus in China is unknown due to the limited number of available sequences, necessitating further research.
